# Direct-acting antivirals used in HCV-related liver disease do not affect thyroid function and autoimmunity

**DOI:** 10.1007/s40618-022-01909-0

**Published:** 2022-09-01

**Authors:** R. Rodia, P. E. Meloni, C. Mascia, C. Balestrieri, V. Ruggiero, G. Serra, M. Conti, M. Loi, F. Pes, S. Onali, A. Perra, R. Littera, F. Velluzzi, S. Mariotti, L. Chessa, F. Boi

**Affiliations:** 1 Endocrinology Unit, Department of Medical Sciences and Public Health, University of Cagliari, Azienda Ospedaliero-Universitaria di Cagliari, SS 554, Bivio per Sestu, 09042 Monserrato, Cagliari Italy; 2 Centre of Liver Diseases, Department of Medical Sciences and Public Health, University of Cagliari, Azienda Ospedaliero-Universitaria di Cagliari, SS 554, Bivio per Sestu, 09042 Monserrato, Cagliari Italy; 3 Unit of Oncology and Molecular Pathology, Department of Biomedical Sciences, University of Cagliari, Cagliari, Italy; 4 Complex Structure of Medical Genetics, R. Binaghi Hospital, ASSL Cagliari, ATS Sardegna, Cagliari, Italy

**Keywords:** Thyroid autoimmunity, Thyroid function, HCV, DAAs, IFN-α

## Abstract

**Purpose:**

It is well known that interferon-α (IFN-α), used for long time as the main therapy for HCV-related disease, induces thyroid alterations, but the impact of the new direct-acting antivirals (DAAs) on thyroid is not established. Aim of this prospective study was to evaluate if DAAs therapy may induce thyroid alterations.

**Methods:**

A total of 113 HCV patients, subdivided at the time of the enrollment in naïve group (*n* = 64) and in IFN-α group (*n* = 49) previously treated with pegylated interferon-α and ribavirin, were evaluated for thyroid function and autoimmunity before and after 20–32 weeks of DAAs.

**Results:**

Before starting DAAs, a total of 8/113 (7.1%) patients showed Hashimoto's thyroiditis (HT) all belonging to IFN-α group (8/49, 16.3%), while no HT cases were found in the naïve group. Overall, 7/113 (6.2%) patients were hypothyroid: 3/64 (4.7%) belonging to naïve group and 4/49 (8.2%) to IFN-α group. Furthermore, a total of 8/113 patients (7.1%) showed subclinical hyperthyroidism: 2/64 (3.1%) were from naïve group and 6/49 (12.2%) from IFN-α group. Interestingly, after DAAs therapy, no new cases of HT, hypothyroidism and hyperthyroidism was found in all series, while 6/11 (54.5%) patients with non-autoimmune subclinical thyroid dysfunction became euthyroid. Finally, the only association between viral genotypes and thyroid alterations was genotype 1 and hypothyroidism.

**Conclusions:**

This study supports evidence that DAAs have a limited or missing influence on thyroid in patients with HCV-related diseases. Moreover, it provides preliminary evidence that subclinical non-autoimmune thyroid dysfunction may improve after HCV infection resolution obtained by DAAs.

## Introduction

Hepatitis C virus (HCV) infection may affect not only the liver but also extrahepatic tissues and organs. This may result both from immunological mechanisms and viral damage in the affected organs. A recent review and meta-analysis [1] confirmed a significant association between HCV infection and both hypothyroidism and serum anti-thyroid autoantibodies (ATA) in naïve patients with viral infection. Among the therapies aimed to eradicate HCV, interferon-α may be considered the first treatment employed since 1980s. However, this treatment showed a low rate of definitive viral clearance [2] and several adverse effects. Thyroid dysfunctions occurred in 4.6–33.3% of pegylated interferon-α (IFN-α)-treated patients, depending on the country considered [3]. IFN-α has been described to induce different types of thyroid damage such as autoimmune and non-autoimmune thyroid alterations, as destructive thyroiditis [4]. In fact, IFN-α can induce both de novo or exacerbate pre-existing HT [5, 6] by increasing immune system cells activity, altering T-regulator and memory *T* cells function, causing the release of IL-6 family cytokines and developing circulating ATA [4]. IFN-α induced HT mostly occurs in female subjects, in the elderly and in patients with other concomitant autoimmune diseases [7]; moreover, as observed in spontaneous HT, it usually persists after therapy withdrawn. On the other hand, IFN-α also has a direct effect on thyroid function, since it inhibits the gene of thyroglobulin, thyroperoxidase and sodium iodide symporter in cultures of human thyrocytes and promotes the apoptosis of thyrocytes. All these mechanisms may account for a spectrum non-autoimmune thyroid damage ranging from subclinical hypothyroidism [8] to transient thyrotoxicosis followed by temporary or permanent hypothyroidism as observed in typical destructive thyroiditis [9]. The course of destructive thyroiditis induced by IFN-α is often mild or subclinical, so that the number of reported cases is likely to be underestimated [10]. Due to the high rate of adverse effects and low therapeutic performance of IFN-α (40%), in these last decades new drugs for HCV treatment have been proposed. In the late 1990s, ribavirin, a purine ribonucleoside analog, were associated with IFN-α and since 2001 to INF-α but this therapeutic approach did not really change the clinical outcome of the disease. Since 2011, the introduction of the first direct-acting antivirals (DAAs), targeting the non-structural proteins of HCV, impress a real revolution in the treatment of this disease, with a greater than 95% improvement of efficacy of these drugs in viral eradication and clinical response of HCV liver disease [11–17]. As reported above, while the impact of IFN-α therapy on thyroid status is well known, few studies exist so far on the potential interference of DAAs on thyroid gland.

With this concept in mind, we prospectively assessed thyroid status (thyroid function, autoimmunity and ultrasound features) in a cohort of HCV patients, subdivided in naïve and previously treated with IFN-α and ribavirin, before and after DAAs therapy.

## Materials and methods

### Patients

**Fig. 1:**
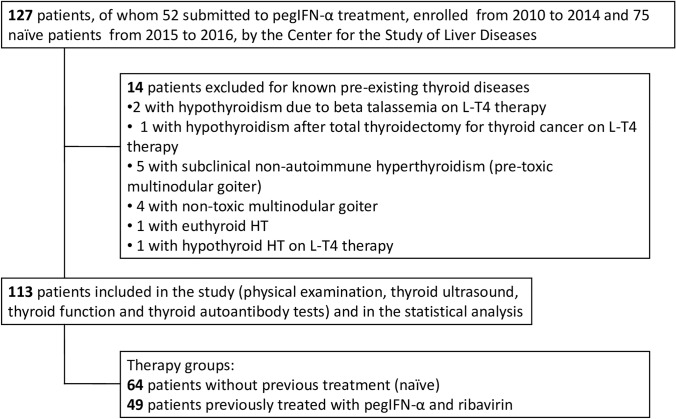
Flow diagram showing protocol followed in patients’ recruitment

This study enrolled a cohort of 127 adult patients with chronic HCV virus infection from the Center Study of Liver Diseases of the University Hospital of Cagliari, Italy. Written informed consent was obtained from each patient after full explanation of the purpose and nature of all procedures used. The Institutional Review Board of University Hospital of Cagliari reviewed and approved the protocol of this study. Thyroid records collected at the moment of the enrollment of all patients, included history of thyroid diseases, treatment with thyroid hormone or anti-thyroid drugs, assessment of thyroid function, anti-thyroid autoantibodies and thyroid ultrasound. According to exclusion criteria (see Fig. 1), patients with thyroid diseases or on thyroid medications, detected before HCV diagnosis, were excluded from this study. As detailed in Fig. 1, we excluded from further analysis 14 cases: 2 patients with hypothyroidism related to beta-thalassemia and 1 with post-surgical hypothyroidism for thyroid cancer, all on L-T4 therapy; 5 with subclinical non-autoimmune hyperthyroidism (pre-toxic multinodular goiter); 4 with non-toxic multinodular goiter; 2 HT (1 euthyroid and 1 hypothyroid on L-T4 therapy). Overall, a total of 113 patients were definitively enrolled and submitted to thyroid evaluation, statistical analysis and subdivided in two groups. The first one, represented by 49 patients enrolled from 2010 to 2014 was previously treated with IFN-α and ribavirin without sustained virological response (median therapy duration 46 weeks), defined as IFN-α group, while the second group of 64 patients, included between 2015 and 2016, was not treated for HCV infection (defined as naïve group). Starting from 2015, all patients were exclusively treated with different DAA-based combination depending on HCV genotype, liver disease severity, and drug availability during the course of the study, such as simeprevir, sofosbuvir, paritaprevir, ritonavir, ombitasvir, daclatasvir, dasabuvir, ledipasvir; median DAAs therapy duration was 16 weeks (range of 12–24). In all patients, thyroid status (assessment of thyroid function, ATA and thyroid ultrasound) was systematically evaluated before and after discontinuation DAAs therapy, with a median interval of 24 weeks (range of 20–32).

### Thyroid function assays

Thyroid hormone and ATA assays were carried out using commercial kits. Thyroid function tests included serum thyroid-stimulating hormone (TSH), free triiodothyronine (fT3), free thyroxine (fT4) (values within the reference range: TSH 0.39–6.16 µUI/ml; fT3 1.4–4.2 pg/ml; fT4 0.8–2 ng/dl), ATA against thyroid peroxidase (TPOAb) and thyroglobulin (TgAb) (TPOAb positive when > 20 AU/ml, TgAb positive when > 4 AU/ml). All these assays were performed by ultrasensitive automatic chemiluminescent method (ELISA Kits DiaMetra S.r.l. Headquarter—Segrate (MI), Italy). TSH-receptor antibodies (TRAb, values within the reference range < 1.86 IU/l) were assayed in hyperthyroid patients by an automated chemiluminescent method (Elecsys^®^ Anti-TSHR, Roche Diagnostics Ltd, Rotkreuz, Switzerland). All the assays were performed in the laboratory of “Duilio Casula” University Hospital of Cagliari. All patients were submitted to thyroid function and autoimmunity assays both before and after DAAs therapy.

### Thyroid ultrasound

Thyroid ultrasound (US) was performed using a Siemens Antares color Doppler system equipment (Siemens, Medical Solutions, Issaquah, WA). According to thyroid ultrasound criteria [18–21], parenchymal echogenicity was classified in normoechoic or hypoechoic pattern (low, moderate and marked, these latter being a typical US feature of thyroid autoimmunity). All patients were submitted to thyroid ultrasound both before and after DAAs therapy.

### Definition of hyperthyroidism, hypothyroidism and autoimmune thyroid disease

Hyperthyroidism and hypothyroidism were diagnosed according to the current guidelines [22, 23]. Overt hyperthyroidism was defined by high serum fT4 or fT3 and undetectable serum TSH concentration, while overt hypothyroidism as low serum fT4 with elevated serum TSH concentration. Subclinical hyperthyroidism was defined as serum TSH below the lower value of the reference range with fT4 and fT3 concentration in the upper part of the reference range; subclinical hypothyroidism was diagnosed by serum TSH above the higher value of the reference range with fT4 concentration in the lower part of the reference range. The diagnosis of autoimmune thyroid disease (HT or Graves’ disease [GD]) was based on the presence of typical US features (hypoechoic pattern for both HT and GD, increased diffuse vascularity for GD), serum positive TgAb and TPOAb (for HT), TRAb (for GD) and increased serum TSH (for HT).

### Statistical analysis

All statistical analyses were performed using Graphpad^®^ Software Inc. (San Diego, USA). Categorical variables were summarized as the counts and percentages (%). Differences between the groups were analyzed by Fisher’s exact test. Statistical significance was defined as *p* values < 0.05.

## Results

### Patient characteristics

As shown in Table 1, among the 113 cases included in the study, 70 (61.9%) were males and 43 (38.1%) were females; the median age of all patients was 60 years (range 31–83). As reported above, patients were subdivided into naïve (*n* = 64) and IFN-α (*n* = 49) groups. The viral genotype was assessed in each patient: 70 patients (61.9%) had type 1, 14 patients (12.4%) type 2, 17 patients (15.1%) type 3 and 12 patients (10.6%) type 4 genotype. As expected, the genotype 1 was found to be the most represented in our cohort of patients and, among this group, 54/70 patients (77.1%) had the subtype b, the most common in Italy and Europe.

**Table 1:** HCV patients’ characteristics and thyroid alterations before DAAs therapy

Patient characteristics	Total	Naive group	IFN-α group
*n*	113	64	49
Male (%)	70 (61.9)	34 (53.1)	36 (73.5)
Female (%)	43 (38.1)	30 (46.9)	13 (26.5)
Median age at enrollment (range)	60 (31–83)	69.5 (31–83)	56 (37–80)
Median DAAs therapy duration (weeks, range)	16 (12–24)	16 (12–24)	16 (12–24)
Normal thyroid function (%)	93 (82.3)	59 (92.2)	34 (69.4)
Total thyroid alterations (%)	20 (17.7)	5 (7.8)	15 (30.6%)
HT − (%)	105 (92.9)	64 (100)	41 (83.7)
HT + (%)	8 (7.1)	0	8 (16.3)
ATA + (%)	8 (7.1)	0	8 (16.3)
Moderate US hypoecogenicity (%)	5 (4.4)	0	5 (4.4)
Marked US hypoecogenicity (%)	3 (2.7)	0	3 (2.7)
Hypothyroidism (%)	7 (6.2)	3 (4.7)	4 (8.2)
Subclinical (%)	3 (2.7)	2 (3.1) ^*δ*^	1 (2.1) ^*δ*^
Overt (%)	4 (3.5)	1 (1.6) ^*δ*^	3 (6.1) ^*γ*^
Subclinical hyperthyroidism (%) ^*δ*^	8 (7.1)	2 (3.1)	6 (12.2)

*HCV* hepatitis C virus, *DAAs* direct-acting antivirals, *HT* Hashimoto’s thyroiditis, *ATA* anti-thyroid antibodies, − absence, + presence, *δ* all ATA−, *γ* all ATA +

### Thyroid alterations before starting DAAs therapy

Thyroid evaluation performed before starting DAAs therapy, showed a high prevalence of thyroid alterations (20/113, 17.7%) in the whole series, being greater (15/49, 30.6%) in IFN-α group (mainly represented by HT), compared to naïve group (5/64, 7.8%); this latter consisted in few cases of non-autoimmune subclinical thyroid dysfunction.

### Thyroid autoimmunity

Before starting DAAs therapy, all the 8 of 113 patients (7.1%) with TPOAb and/or TgAb positive (indicated as ATA + in Table 1) belonged to IFN-α group; 5 of them showed moderate US hypoechogenicity and were euthyroid, while the remaining 3 had marked US hypoechogenicity with overt hypothyroidism; all fulfilled the diagnostic criteria of HT. As expected, the percentage of HT was higher in female (5/43, 11.6%) compared to male (3/70, 4.3%, *p* = 0,25).

### Hypothyroidism

As reported in Table 1, before starting DAAs therapy, hypothyroidism was detected in 7 of 113 patients (6.2%). Four/7 hypothyroid patients belonged to IFN-α group, 3 of them had overt autoimmune (ATA-positive, hypoechoic gland) hypothyroidism, while only one had subclinical non-autoimmune (ATA-negative, normoechoic gland) hypothyroidism. Among the 3 non-autoimmune hypothyroid patients of the naïve group, 1 had overt hypothyroidism and 2 had subclinical hypothyroidism. All patients with overt hypothyroidism underwent L-thyroxine replacement therapy, while cases with subclinical hypothyroidism were only followed up. Finally, as expected, a higher prevalence of hypothyroidism was observed in female (7/43; 16.3%) than in male (0/70, 0%; *p* = 0,0008).

### Hyperthyroidism

Before starting DAAs therapy, subclinical hyperthyroidism, was found in 8 of 113 patients (7.1%) and 6 of them belonged to IFN-α group, while the remaining 2 were in the naïve group, see Table 1. No overt hyperthyroidism and ATA positivity (TRAb included) was observed in all cases. Mild US alterations represented by a slight reduction of thyroid volume and vascularization was observed in almost all cases, especially in IFN-α group patients. In 2 of these patients, thyroid scintiscan showed reduced uptake, leading to the presumptive diagnosis of destructive thyroiditis.

### Thyroid alterations and viral genotypes

No significant difference in HT prevalence was found between genotype 1 (5/70 patients, 7.1%) compared to the other genotypes considered together (3/43 patients, 7%). In particular, HT was observed in 2/17 of genotype 3, and 1/12 of genotype 4; no case of HT was found in genotype 2, probably due to the low number (4 of 14) of IFN-α treated patients. Similar findings were observed for subclinical hyperthyroidism, without significant differences between genotype 1 (3/70 patients, 4.3%) and the other genotypes considered together (5/43 patients, 11.6%, *p* = 0,25), with 2/14 for genotype 2, 2/17 for genotype 3 and 1/12 for genotype 4. In contrast, a significant difference in prevalence of combined autoimmune and non-autoimmune hypothyroidism was found between genotype 1 (7/70 patients, 10%) compared to the other genotypes (0/43, *p* = 0.042), see Table 2.

**Table 2:** Thyroid alterations and HCV genotype

Patient genotype	Total	Naive group	IFN-α group	HT	Hyperthyroidism	Hypothyroidism	*p*
*n*	113	64	49	8	8	7	
Genotype 1 (%)	70 (61.9)	45 (70.3)	25 (51)	5 (62.5)	3 (37.5)	7 (100) *	*p = 0.042 vs all the others genotypes
Genotype 2 (%)	14 (12.4)	10 (15.6)	4 (8.2)	0	2 (25)	0	
Genotype 3 (%)	17 (15.1)	4 (6.3)	12 (24.5)	2 (25)	2 (25)	0	
Genotype 4 (%)	12 (10.6)	5 (7.8)	8 (16.3)	1 (12.5)	1 (12.5)	0	

*HCV* hepatitis C virus, *HT* Hashimoto’s thyroiditis

### Thyroid alterations after DAAs therapy discontinuation

Interestingly, after DAAs therapy discontinuation, no new cases of autoimmune thyroid disease were found in both groups, while the 8 HT patients of IFN-α group, maintained unaltered their clinical features. Furthermore, no new cases of both hypothyroidism and hyperthyroidism were observed after DAAs withdrawn in the whole series.

**Fig. 2:**
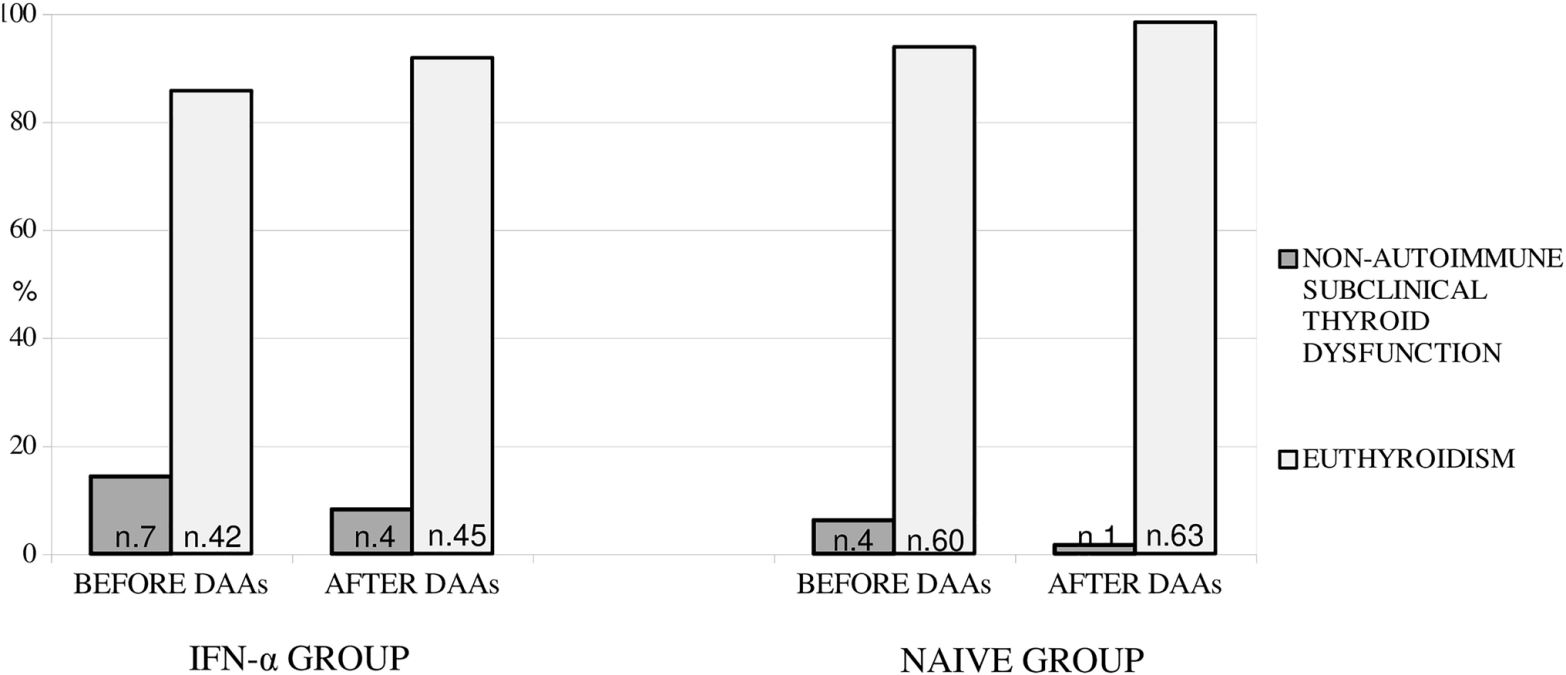
Prevalence of non-autoimmune subclinical thyroid dysfunction in HCV patients of IFN-α and naïve group before and after DAAs therapy. The number of patients with thyroid dysfunction are reported in each column of the figure

Finally, as shown in Fig. 2, among the 11 patients (4 of naïve group and 7 of IFN-α group) with non-autoimmune subclinical thyroid dysfunction, 6 (54,5%) became euthyroid after DAAs discontinuation: 3 belonging to IFN-α group (1 hypothyroid and 2 hyperthyroid) and 3 to naïve group (2 hypothyroid and 1 hyperthyroid).

## Discussion

It is well known that HCV patients may develop various thyroid alterations related to HCV infections, as observed in a significant proportion of HCV naïve patients, and to IFN-α treatment [7]. Recently, it has been shown that HCV could directly infect the human thyroid cell, suggesting its possible direct role in thyroid dysfunction. However, several studies indicated that thyroid alterations may be mediated by immunological mechanisms, rather than a direct effect of HCV infection [1]. The impact of IFN-α treatment on thyroid gland is well known and is mediated by numerous immunomodulatory effects, such as immune responses activation. According to these evidences, we found higher prevalence of thyroid alterations in our series compared to European [24, 25] and Sardinian [26, 27] general population; as expected, we also observed a higher prevalence of these alterations in IFN-α group (30.6%) compared to naïve (7.8%) group. The low prevalence of ATA and TH (7.1%) observed in our series, was not completely unexpected on the basis of an our previous study [27], in which we found no evidence for epidemiological association between circulating ATA and antibodies to HCV in Sardinian general population. Despite this evidence, similarly to the epidemiological data [24, 27, 28], a higher rate of HT and hypothyroidism was observed in female (11.6 and 16.3%) compared to male (4.3 and 0%), respectively.

Until now, scanty studies have assessed the prevalence of thyroid dysfunction related to HCV treatment with DAAs and conflicting results are so far reported. In particular, in a study of Wahid et al. [29], performed on a limited number of patients (37 patients on sofosbuvir and 26 IFN-α treated patients), was reported high prevalence of hypothyroidism in both groups, with 18.9% observed in sofosbuvir group. In a more recent study, Wahid et al. [30] confirmed a high risk of hypothyroidism in two groups of HCV patients treated with different DAAs drugs regimens (sofosbuvir + IFN-α + ribavirin and sofosbuvir + daclatasvir + ribavirin), with higher prevalence of hypothyroidism in the first DAAs group. Eletreby et al. [31] studied a very large number of HCV naïve patients (13,402 patients), detecting at baseline a total of 21.1% of hypothyroidism. Interestingly, in a subgroup of patients (*n* = 236) of this series, TSH re-evaluated after DAAs discontinuation and HCV disease resolution, resulted improved in about 80% of cases. However, this study did not assess autoimmune and morphological thyroid features (thyroid autoantibodies and thyroid ultrasound) neither at baseline nor after DAAs therapy discontinuation. Based on these contradictory data, to clarify the impact of DAAs drugs on thyroid gland, we systematically evaluate thyroid function, thyroid autoantibodies and thyroid US before and after a full cycle of DAAs therapy both in naïve and IFN-α with ribavirin previous treated HCV patients. For the first time, our data suggest a limited or missing influence of DAAs therapy on thyroid function and autoimmunity of patients studied, as deduced by the unchanged number of HT patients in IFN-α group and the absence of new cases observed in both groups. Based on these evidences, we speculate that, at difference of IFN-α treatment, DAAs did not interfere on the immune system and did not affect thyroid function.

Finally, according to the literature, we also observed a high proportion of patients with non-autoimmune subclinical thyroid dysfunction (54.5%) that became euthyroid after DAAs discontinuation. We admit that, in patients with a transient subclinical hyperthyroidism, the recovery of thyroid function, might also be explainable with the course of nonthyroidal illness syndrome, related to HCV disease. In addition, the impact of biological variation in TSH should be considered as this influence thyroid function classification. The diagnosis of subclinical hyperthyroidism was also sustained by the detection of serum FT3 in the upper part of the reference range (although this analyte should be evaluated cautiously, since it may be influenced by binding proteins modification and assay-variations); moreover, the reduction of thyroid scintiscan uptake observed in two cases, lead to the presumptive diagnosis of destructive thyroiditis. This phenomenon could be explained by the achievement of a sustained virological response after DAAs, that appears to improve thyroid dysfunction, as recently described to Eletreby R et al. [31]. According to a recent review and meta-analysis [1] and based on clinical outcome of our patients with non-autoimmune subclinical thyroid dysfunction, we agree that HCV infection might play a pathogenic role, and as reported by Hammerstad et al. [32], thyroid alterations may result both from indirect immunological mechanisms and direct viral damage.

## Conclusions

Our study conforms to other studies about the high prevalence of thyroid alterations related to HCV infection and IFN-α treatment, that suggests periodic evaluation of thyroid status both in naïve patients and in those under therapy. Moreover, for the first time, the present study supports evidence that direct-acting antivirals have a limited or missing influence on thyroid function and autoimmunity, both in naïve and IFN-α previously treated patients up to several weeks after DAAs discontinuation. Finally, we provide evidence that non-autoimmune subclinical thyroid dysfunction potentially induced by HCV infection may improve after viral clearance with DAAs therapy. Further larger multicentric controlled studies are needed to confirm the clinical relevance of our research.

## Data Availability

All data generated or analyzed during this study are included in this published article.
